# Two steps back, one step forward: reconstructing the dynamic Danube riverscape under human influence in Vienna

**DOI:** 10.1007/s12685-013-0076-0

**Published:** 2013-07-03

**Authors:** Severin Hohensinner, Christoph Sonnlechner, Martin Schmid, Verena Winiwarter

**Affiliations:** 1 Institute of Hydrobiology and Aquatic Ecosystem Management (IHG), University of Natural Resources and Life Sciences Vienna (BOKU), Max-Emanuel-Str. 17, 1180 Vienna, Austria; 2 Municipal and Provincial Archives of Vienna, Rathaus, 1082 Vienna, Austria; 3 Centre for Environmental History (ZUG), Alpen-Adria University Klagenfurt, Schottenfeldgasse 29, 1070 Vienna, Austria

**Keywords:** Reconstruction, Historical GIS, River, Historical change, Vienna, Danube

## Abstract

As part of an interdisciplinary project on the environmental history of the Viennese Danube, the past river landscape was reconstructed. This article describes the different types of historical sources used for the GIS-based reconstruction, the underlying methodological approach and its limitations regarding reliability and information value. The reconstruction was based on three cornerstones: (1) the available historical sources; (2) knowledge about morphological processes typical for the Austrian Danube prior to regulation; and (3) the interpretation of past hydraulic measures with respect to their effectiveness and their impact on the river’s behaviour. We compiled ten historical states of the riverscape step-by-step going backwards in time to the early 16th century. After one historical situation had been completed, we evaluated its relevance for the temporally younger situations and whether corrections would have to be made. Such a regressive-iterative approach allows for permanent critical revision of the reconstructed time segments already processed. The resulting maps of the Danube floodplain from 1529 to 2010 provide a solid basis for interpreting the environmental conditions for Vienna’s urban development. They also help to localise certain riverine and urban landmarks (such as river arms or bridges) relevant for the history of Vienna. We conclude that the diversity of approaches and findings of the historical and natural sciences (river morphology, hydrology) provide key synergies.

## Introduction

This article presents a regressive-iterative approach for reconstructing historical landscapes using a geographical information system (GIS). In historical research, regressive methods moving step-by-step back in time from a better known later situation were already applied by Seebohm (1883) or by Bloch (1931) for the reconstruction of medieval agrarian landscapes in France and have been used since then within historical research (e.g. Forschungsinitiative Umweltgeschichte 1999). The method presented here takes a similar approach. In this study, we integrated three types of evidence: (1) numerous historical sources, both textual and cartographic, (2) analysis of fluvial processes that were typical for the Austrian Danube before regulation, and (3) assessment of river engineering measures of the past with regard to their effectiveness and their impact on river behaviour. This approach constitutes a temporally regressive method (in the sense of Marc Bloch), but comprises iterative work steps to refine the results for more recent time situations.

The aim to “reconstruct” the historical development of a river landscape true-to-life is a priori doomed to fail. The preserved information is too fragmentary; the available sources are too different in type and content. Some sources are more resistant to the reconstruction of past riverscapes than others. Historical sources (maps, plans, documents, etc.) were not created for use in a GIS, they were produced for particular reasons and thus always reflect how the riverscape was perceived. They reflect the interests and motives of their producers and recipients and are therefore only fragments of a historical state. The methodological quest is to determine which fragments can be useful for the reconstruction. The challenge is greater in a landscape that has undergone incessant change, as was the case with the historical Danube near Vienna. It has to be borne in mind that all historical relicts from the Danube’s history are sources for the changing perception of that river. Historians ask for the motivation and interests that were important in the making, using and keeping of their sources (Clanchy 1993). Approaching a source from an environmental history viewpoint means interpreting it both as an expression of changing biophysical relations to the environment and of changing cultural attitudes, ideas and ideals about nature. Interpreting it requires integrative methods including source critique. An interdisciplinary team’s different perspectives are helpful in this endeavour. So our project team included historians from the Centre for Environmental History Vienna (Alpen-Adria University Klagenfurt) and the Municipal and Provincial Archives of Vienna, and fluvial morphologists from the University of Natural Resources and Life Sciences Vienna (BOKU).

Reconstructing riverscapes over decades or even centuries better approximates the former situations than the reconstruction of a single point in time. In recent years, GIS-based studies of historical fluvial morphology were conducted over the long term to reveal the causes of past channel changes and floodplain degradation (Gurnell et al. 1994, 2005; Marston et al. 1995; Kiss et al. 2008). Some historical studies specifically focus on the spatial distribution of riverine habitats and the land cover of riverscapes. In Europe, such investigations were conducted on several French rivers (e.g. Girel et al. 1997; Kondolf et al. 2007), the current Slovak Danube (Pisút 2002), the lower Rhine (Schoor et al. 1999; Wolfert 2001), the Dyje River, Czech Republic (Skokanova 2008) and on several English rivers (Lewin 2010). Outside Europe, comparable studies exist for e.g. the upper Mississippi (de Jager et al. 2011) and the Sacramento River, California (Greco et al. 2007). In ecology and environmental history alike, GIS techniques have often been deployed to reconstruct past land cover and land use changes based on historical maps such as cadastral maps. These include, amongst others, the Baltimore–Chesapeake region (Foresman et al. 1997), the American Great Plains (Cunfer 2008), the Rocky Mountains (Aspinall 2004), Southern Germany (Bender et al. 2005; Schuppert and Dix 2009), the Tisza River in Hungary (Hegedüs and Duray 2009) and several Austrian villages and rivers (Forschungsinitiative Umweltgeschichte 1999; Haidvogl 2008). Knowles (2002) has already demonstrated the potential of GIS techniques for historical research.

The GIS-based river and floodplain reconstruction method developed by Hohensinner was first applied to identify historical alterations of the Danube riverscape in the Austrian Machland region 160 km upstream from Vienna (Hohensinner 2008; Hohensinner et al. 2011) and in the Lobau floodplain directly downstream from Vienna (Hohensinner et al. 2008). Here, we present a refined method to reconstruct the Viennese Danube over 500 years. The study site refers to the extents of the recent alluvium of the Danube (post-glacial). Since up- and downstream river sections are basic for understanding local fluvial changes, it is almost 18 km long (compare Fig. 7). We produced two GIS databases for the reconstruction: a database of historical river engineering measures and a second one with more than 200 georeferenced maps. Both were combined with a newly compiled flood database to understand both the natural and the human causes of river morphology change in and around Vienna.

## The integration of historical sources into the reconstruction

The Viennese Danube’s historical heritage is voluminous. Various archives house thousands of maps, plans and topographical views, along with thousands of pages of text from the 14th century onwards. GIS-based landscape reconstructions of other rivers and Danube sections usually focus on the last 200–300 years (Hohensinner et al. 2011). In Vienna, however, the abundant sources allow for a reconstruction covering almost 500 years in total. At the same time, this abundance makes reconstruction more difficult. The individual sources often show contradictory information about a certain historical state of the riverscape or the implemented hydraulic constructions. As such, they have to be critically assessed. We reconstructed the Viennese riverscape primarily based on historical maps, plans and topographical views. In addition, we used written sources to validate the information from the maps and to add details not covered by the topographical sources. Maps and plans produced after 1700 generally show a more consistent geographical projection and a higher level of detail. After 1800, cartographic techniques improved, in particular when cadastre maps were used as a basis for city maps or regulation plans. The following sections demonstrate how we used these diverse sources for an integrative reconstruction of a riverscape.

### The 16th and early 17th centuries: the key phase to understand the riverscape

**Fig. 1:**
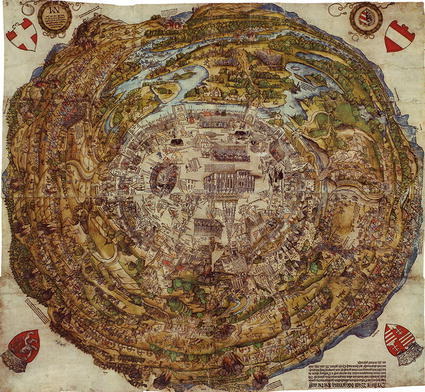
Vienna’s surroundings during the first siege by the Ottoman army in 1529 (Meldeman 1530, Wien Museum, Sign. 48.068)

The oldest topographical views that have proved useful for reconstruction show the city and its environs during and a few years after the first siege of Vienna by the Ottoman army in 1529. Among the most important of these is the so-called “Meldeman-Plan” published by Niclas Meldeman in 1530.^1^ The master drawing for this illustration was created by an anonymous artist (possibly H. Sebald Beham), who reportedly lived in Vienna during the Ottoman siege (Meldeman 1530; Düriegl 1980). It reveals valuable information on floodplain topography, riverine structures, settlements, land cover, bridges and roads (see Fig. 1).

Of particular interest are the spatial arrangement of the diverse river arms and floodplain water bodies and the indicated cut- and accreting banks. We can safely assume that the latter features were painted with no particular interest on the part of the map-makers, as they are not central to the depiction. Such riverine elements not only reflect the morphological state of the riverscape at a specific point in time, they also allow conclusions to be drawn about its configuration several years before and potentially after the point of depiction. For example, the steep cut banks together with abandoned river arms in front of the image point to a former dynamic river arm that eroded the margins of an older river terrace several years or a few decades earlier; the time span depends on the river type. Similar landscape structures may also derive from the extraction of clay for mud brick production, which probably has occurred at some sites in the example described (Suess 1862).

Interpreting such sources necessitates consideration of the aims of their creators. The picture from 1530, like many others we used in our study, expresses fears and hopes connected to the Danube in early modern times. For more than one and a half centuries, between 1521, when Belgrade was captured by the Ottomans and 1683, when Vienna was besieged a second time, two-thirds of the Danube River was controlled by the Ottoman Empire. This influenced representations and perceptions of the Danube on both sides, even if we see only the Habsburg perspective in our sources. The Meldeman plan’s main purpose was to capture the theatre of war. The riverscape was included because it was an important part of the battlefield. Some landscape and riverine structures may have been omitted to emphasise details that were more important to illustrate the course of the siege, but we assume that the status of the river—the abundance of small channels and islands—was depicted plausibly, as it was part of the battle site being represented.

**Fig. 2:**
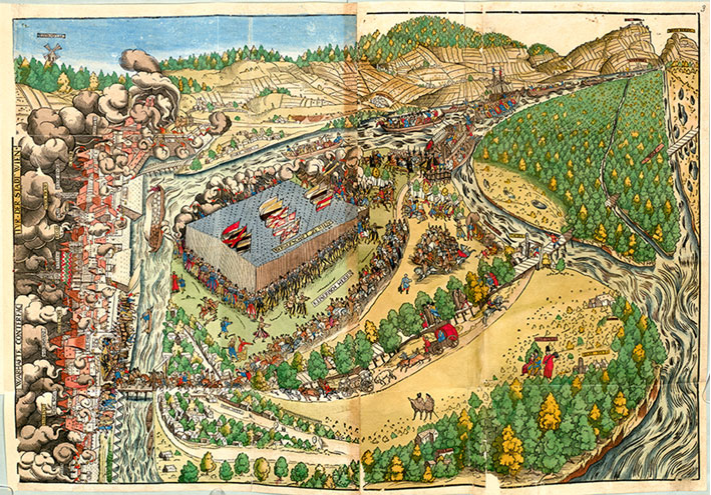
“WARHAFTE CONTERFACTVR DER STADT WIEN” (“True delineation of the city of Vienna”). The woodcut from Hans Mayr, published by Stainhofer (1566), shows the festive arrival of Emperor Maximilian II in Vienna in 1563. On the *left margin*: the side arm *Wiener arm*, later called *Donaukanal*; in the *middle*: the *Tabor arm*, main branch until c. 1565 with the *Tabor* toll gate; on the right margin/north from the city: *Wolf arm*, side arm until c. 1565, later main arm. (Bayerische Staatsbibliothek München, Sign. Rar. 250, fol. 3r)

From 1560 onwards, several topographical sources providing relevant information about the status of the riverscape are available. In the 16th century, imperial celebrations were a favoured subject of topographical views (e.g. Francolin and Hofhalter 1561). In such pictorial sources, the Danube riverscape provides the arena for the display of imperial power. On 16 March 1563, Emperor Maximilian II returned from his coronation in Frankfurt to Vienna. Three years later, Caspar Stainhofer published an illustrated description of the imperial entry, accompanied by an illustration entitled “WARHAFTE CONTERFACTVR DER STADT WIEN” (“True delineation of the city of Vienna”, Stainhofer 1566; Fig. 2).^2^

In the report, even the river itself attests to its happiness regarding the emperor’s return (“… der wasserfluß, Mit freud der gibt auch zeugnuß”). Stainhofer’s report points to one meaning that riverscapes had for early modern European societies: They were places where social order was manifested and where those in power could demonstrate their control over nature (cf. Winiwarter 2006). The riverine structures depicted provide valuable information for the reconstruction in addition to the location of the main Danube branches. Both the sinuous course of the middle arm (*Tabor arm* due to the toll building at the southern bank called *Tabor*) and the distinct point bar along its northern bank indicate a continuing meandering process (Fig. 2). At the northern arm (*Wolf arm*), the extension of the water-covered area in relation to unvegetated gravel bars suggests a former main arm recently abandoned by the Danube. Combined with information from written sources, however, the picture is different: It reflects the state of amplified channel dynamics due to a shifting of the main current from the *Tabor arm* to the *Wolf arm*.^3^ In addition, the described illustration provides interesting information on the location of bridges and roads. In this respect, several archival files and a description of the bridges in 1547 by Wolfgang Schmeltzl (1548) proved the illustration to be highly authentic.^4^ This example shows that the depicted landscape structures must be critically questioned and reconciled with other sources. From comparing dozens of maps and topographical views, we have gained the impression that the long-term residence of a mapmaker in Vienna tends to be associated with depictions that are more useful for reconstruction purposes. The woodcutter for the illustration of the ceremony, Hans Mayr from Leipzig, was active at the Viennese court in the 1560s (Wünsch 1914).

In the late 16th century, conflicts regarding property borders gave rise to a series of topographical sources that contain evidence on details of the Danube riverscape. These conflicts are associated with the above-mentioned major shifts of the main river arms from c. 1560 onwards. Erosion of floodplain areas intensified and new islands formed. Two major landowners, the monastery of Klosterneuburg and the Burghers’ Hospital contested the ownership of land that was fluid (Sonnlechner et al. 2013, in this issue). In order to document the state prior to the conflict, both sides produced views and a map designed to support their arguments.^5^ They all were previously dated to 1632. Based on a comparative analysis with sources showing younger and older states of the riverscape, we surmise that four views in fact reflect the riverscape’s status in c. 1570/80 and not in 1632. A comprehensive review of historical documents and literature based on the indicated locations of bridges, roads and the altered toll buildings (*Tabor*) proves that assumption to be correct. Only the map that has been totally disregarded so far can be related to 1632.^6^ Our research revealed that it is the oldest map that covers the Viennese floodplain in the plan-view. The example shows that the archival dating of historical sources can yield misleading conclusions in respect of the riverscape’s state at a certain point in time.

Taken together, the various historical sources document a major rearrangement of the Danube channel network. In order to conclude whether identified fluvial dynamics reflect the river’s typical behaviour rather than an exceptional hydromorphological state, climatic changes and related flood regimes also have to be considered (Howard 1996; McCarney-Castle et al. 2011; Macklin et al. 2012). For example, increasing runoff generally leads to channel straightening and profile widening. Together with augmented sediment loads, it additionally fosters the transformation from meandering to braiding (Nanson and Knighton 1996; Marti and Bezzola 2004). The major channel shifts at the Viennese Danube therefore have to be interpreted against the background of the *Grindelwald Fluctuation*, the first extreme phase of the *Little Ice Age* from the 1560s to the 1620s (Pfister 1980, 2007; Behringer 1999; Hohensinner et al. 2013, in this issue). Accordingly, the period from the mid to late 16th century must be considered as the key phase to understand the evolution of the riverscape, the intentions of discussed/implemented regulation measures and, consequently, the interpretation of historical sources in the following centuries.

**Fig. 3:**
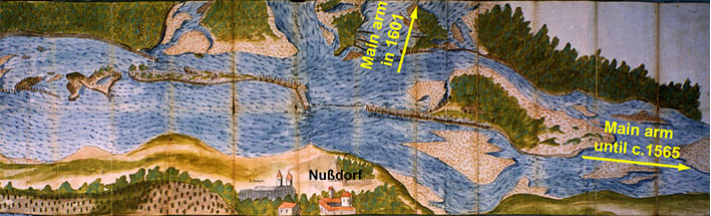
Danube River near Nußdorf (upstream from Vienna) in 1601 (Thomas Clausniez 1601, OeStA, AVA—FHKA, Kartensammlung, Sign. F 245)

The earliest river engineering plan found so far originates from hydraulic engineer Hans Gast from 1598. In 1601, Thomas Clausniez (see Fig. 3) and Maximilian Saurer drew additional plans.^7^ The intensified planning activities can be explained by the channel changes that culminated in 1565/1566.

River engineering plans must be interpreted with caution because many of the depicted hydraulic constructions were never implemented or were realised differently. The plans served as a basis for the discussion of the technical feasibility or the required costs by the authorities in charge (Sonnlechner et al. 2013, in this issue). Several plans together with the associated manuscripts contain quantitative data such as the lengths of planned structures, distances in relation to river banks, bifurcations or already existing constructions. This helps us to estimate the widths of river arms and islands; selected hydraulic structures can be used as landmarks to refine the positioning of riverine and human structures. Also the nomenclature of river arms and the constructions proved to be very useful. In particular, when existing structures (spur dikes, training walls) are identified by the names of their constructors. This helps to determine the position of buildings that were created several years or even decades earlier than the mapped situation. In rare cases, the plans themselves specify whether the constructions depicted were only projects or actually existed. Identifying the implemented hydraulic measures requires the comparative analysis of several plans showing the situation at the same time or within a short time span; research on the respective manuscripts can help to clarify these uncertainties.

**Fig. 4:**
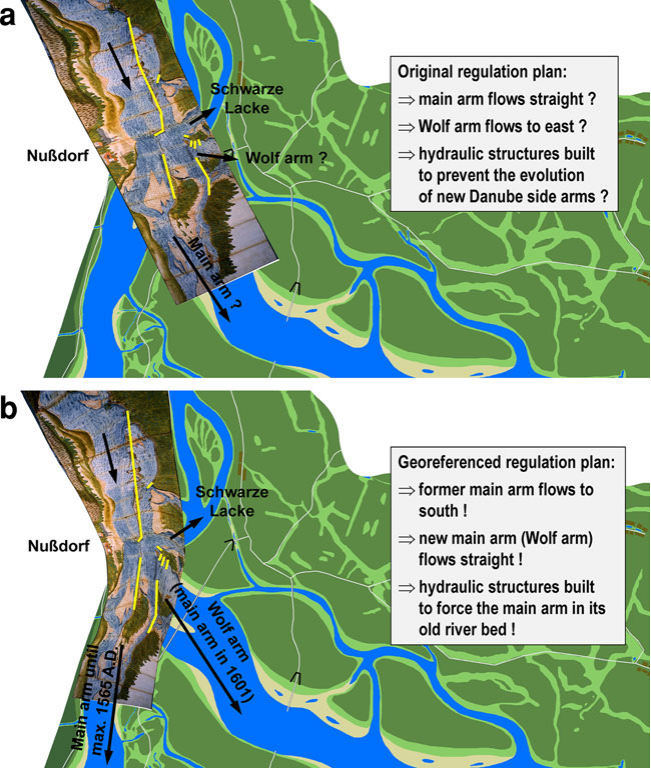
**a** Conclusions on the configuration of the river landscape and the purpose of the hydraulic constructions without considering the true topography; **b** conclusions when considering the true topography (*yellow lines* hydraulic constructions; the *background* shows the situation in 1570)

Comparative analysis of maps shows that representations of the river landscape up until the first half of the 17th century were based on the cartographers’ perception of the relative importance of the individual elements of the riverscape. The relative importance of features depends on the historical context that has to be investigated to assess the maps. Maps from Nußdorf at the Danube upstream of the historical centre of Vienna show a major arm in 1600 mapped as a straight line, emphasising it as the main arm of the Danube. In fact, its course was actually strongly sinuous at that time and it was no longer the main arm of the Danube. Due to the vital importance of this former main arm in supplying the city of Vienna, it continued to be depicted as the central element in the maps, whereas the actual main arm was represented as a minor side arm on the edge of the map (compare Fig. 3). Maps from Clausniez and Saurer (both from 1601) provide indications: they identify the confluences of small mountainous tributaries and the bifurcations of large river arms along the Danube’s course. Using the confluences as landmarks in a GIS, the historical maps can be easily georeferenced. As we came to understand this situation, our conclusions about the configuration of the river landscape in 1600 and, consequently, about the purpose of the indicated hydraulic constructions were profoundly altered (compare Fig. 4a and Fig. 4b).

The historical literature on the Danube and the *Donaukanal*, a side arm of the Danube which served as the only waterway giving access to the historical city centre, over the past 250 years has followed the misleading representation in the early cartography of the Danube, without addressing the former situation as it was.^8^

### The late 17th and 18th centuries: new types of historical sources

The increased threat posed by the Ottoman army and the second siege of Vienna in 1683 gave rise to numerous maps and views that illustrate acts of war. Most of them focus only on the historical city centre or show the riverscape in a generalised manner. The riverine structures were mostly copied from older drawings, which can be identified as sources. Hence, little information about the past configuration of the riverscape can be extracted from the great number of 17th/18th century maps and illustrations. The most important exception is a map designed by Colonel Giuseppe Baron Priami in 1663 for the improvement of Vienna’s fortifications. It can be considered as the first map of the Viennese riverscape, which is depicted in a geographically largely correct manner (Mohilla and Michlmayr 1996; Opll 2004).^9^ Even more interesting are several river regulation plans that were drawn after the siege from 1686 onwards, in particular the famous work of the Italian cartographer Leander Anguissola from 1688 and a newly found map by Hoffmann von Anckherskron et al. dating from 1700.^10^ Compared to older plans and maps, both maps show large areas of the Viennese riverscape in a regular map projection. Problems remain: In Anguissola’s map, the differentiation between planned and existing hydraulic structures is not always clear and the map was modified at a later date to adapt it to the changed conditions of the riverscape. For example, a new cut-off channel at the *Donaukanal* excavated in 1700–1703 and bridges built in 1704 were later added, so it could serve as the basis for proposed hydraulic constructions in 1712 (Slezak 1977).^11^ The map from Hoffmann von Anckherskron et al. (1700) can be considered as the oldest Viennese river engineering map with a high degree of position accuracy and an outstanding level of detail. It was produced as a planning basis for the construction of a new course for the upper *Donaukanal* and so far it has never been described in the historical literature. It even shows minor relicts of past hydraulic structures below the low water level and several transects through the river arms. The map provides a sound reference for the localisation of hydraulic structures built in the late 17th century of which—until now—we only partly knew about from written sources.

In the first half of the 18th century, the number of topographical sources substantially increased, and from the late 18th century a great variety of different types are available. At that time, topographical views and maps produced for commercial purposes gained considerable public attention. Several of these maps were created on the basis of the well-known city map from Leander Anguissola and Johann Jacob Marinoni in 1704 and published under the title “Accuratissima Viennae Austriae Ichnographica Delineatio” (“Most accurate plan of Vienna in Austria”) in 1706.^12^ True to its title, the plan shows the city with its fortifications and with the growing suburbs, which at that time had already spread into the riverine landscape—namely the *Leopoldstadt* on the large island confined by the Danube and the *Donaukanal* (here called the *Neuer Canal*; Haidvogl et al. 2013, in this issue). In this plan the Danube itself is at least as important as the urban settlement. The main contemporary intention of the plan was to show the newly strengthened fortifications of the Habsburg residence. An additional second ring of fortification walls and ramparts (called the *Linienwall*) had been built in 1704 to better protect the residence. This plan makes clear that the Danube was an essential part of the city’s fortification system. To the northeast, the Danube was Vienna’s fortification. In the floodplain, only parts of the *Leopoldstadt* and the head of the only bridge crossing the main arm of the Danube were fortified with man-made structures. In later editions of the map up to c. 1785, only the settlement areas within the town were updated; the riverscape was depicted as unchanged, a pretence that the riverscape had been stable over decades. However, the comparison with the famous “Jagdatlas Kaiser Karls VI.” (“Atlas of imperial hunting grounds”) produced by J. J. Marinoni between 1726 and 1729 reveals that the riverscape had experienced substantial alterations since 1704.^13^ This map series is the first geometrically coherent cartographic source that also covers areas remote from the historical city centre (Marinoni 1751).^14^

In the 18th century, the growth of the city also gave rise to new regulation projects. Thus hundreds of hydraulic construction plans were generated, but many show constructions never implemented. Numerous of these plans were compiled by the hydraulic engineer Johann Sigismund Hubert, who constructed the first larger flood protection scheme for Vienna. Since most plans only refer to minor regulation works, many of them have never been described in the literature. In this case, only the comparative analysis of the numerous plans with written sources can help clarify which works were actually realised.

The “First Military Survey” (“Josephinische Landesaufnahme”) 1769–1785 and the “Second Military Survey” (“Franziszeische Landesaufnahme”) 1806–1869 are the first map series covering the whole Habsburg Empire.^15^ The maps of the Vienna region reflect the situation in 1780 and 1809, respectively. With respect to the level of detail, both maps are wanting, and several details like the hydraulic structures at the inflow of the *Donaukanal* were added later to the “First Military Survey”. These updates led to confusion about the correct years of construction of several hydraulic structures and of the infrastructure (roads, bridges) in the floodplain. Though the military surveys provide an impressive overview of the riverscape and its environs, one has to strive to find the individual construction plans or land property maps. Accordingly, we could use the “First Military Survey” only as a rough topographic basis for the reconstruction of the riverscape in the whole study site in 1780. For further refinement, we used numerous other written and cartographic sources.

### The 19th century: preparing the Danube regulation

In contrast to the military surveys, the first Danube-wide map series from 1816 to 1817, known as the “Lorenzo–Karte” (scales 1:7,200 and 1:28,800) after its creator Christophorus de Lorenzo, provides detailed information about the configuration of the whole riverscape and is useful due to its consistent projection.^16^ It contains several hydromorphological data, like terrain heights, channel slopes and flow velocities. In combination with the Viennese cadastral maps (“Franziszeischer Kataster”, scale 1:2,880), which were produced between 1817 and 1825, a good localisation of the riverine structures shown in the “Lorenzo–Karte” can be achieved.^17^ Though the cadastral maps offer a high level of detail, small riverine structures are reflected poorly. The tax cadaster focuses on plot boundaries and land uses, therefore some sheets of the map omit structures like gravel bars and small water bodies. But taken together, the “Lorenzo–Karte” and the cadastral maps provide numerous landmarks for the correct positioning of riverine and human structures in the earlier time segments and, thus, can be considered as a “backbone” of the chronological GIS-reconstruction.

From the 1830s onwards, plans, maps and topographical views are abundant. These include river regulation plans, navigation maps, military surveys, administrative maps, city maps, etc. published by governmental authorities, by the *Danube Regulation Commission* or by companies for commercial use. One map series of the Danube River that is commonly used to illustrate the former state of the Danube riverscape is the well-known “Pasetti–Karte”.^18^ It was generated between 1857 and 1867 under the direction of Florian Ritter von Pasetti and covers the Danube from Passau to the Iron Gate. Despite its popularity it is poorly suited for direct reconstruction. But it shows detailed information about different types of river banks, channel slopes, the state of river engineering works, infrastructure in the floodplain, etc. These data help to determine years of construction and to estimate the potential consequences of the hydraulic structures on fluvial processes, e.g. deflection of the current by a new training wall and downstream bank erosion, terrestrialization processes in dammed up side arms and behind dikes.

The discussion about a comprehensive Danube regulation for Vienna triggered the preparation of numerous plans, maps and technical reports from 1849 onwards. Besides the *Danube Regulation Commission*, several professionals, stakeholders and individuals tried to gain public attention by the publication of their own studies and plans for the Danube regulation. One of the most fascinating maps was created in 1849: the first altitudinal survey of the whole Viennese riverscape. It was elaborated under the direction of Valentin Streffleur as basis for the large regulation programme.^19^ Besides hydromorphological data and land cover, it shows small water bodies and minor depressions in the floodplain terrain. It provides an inestimably valuable source for the identification and localisation of river arms that existed decades or even centuries earlier. Based on this survey, we generated a digital terrain model in 2007 that served as a main basis for the reconstruction works in the current study (Herrnegger 2007; Hohensinner et al. 2008). From the period when the comprehensive regulation programme was finally accomplished (1870–1875), a multitude of historical data is available, from very detailed technical plans and reports to illustrative maps for the interested public, which are less useful for reconstruction.

## Reconstructing the dynamic Danube riverscape

As the first step of the reconstruction, we evaluated the available historical sources with regard to their relevance for the project. From more than 1,000 historical maps, plans and topographical views, we scanned more than 400 sources and georeferenced more than 200 with ESRI ArcGIS 10. For that, we recorded the type and suitability of most of the sources in a database. Besides general attributes, we identified and coded the accuracy of relative position, the level of detail and the mapped elements of interest such as riverine and floodplain structures, settlements, infrastructure, additional hydromorphological information, etc. This allowed an initial classification of their usability. In the course of reconstructing the Viennese riverscape, the historical sources provided the most important, but not the sole basis.

### River morphological background

Most rivers in Europe have fundamentally changed from their natural status. Lewin (2010) concludes that most of the larger medieval lowland rivers in England seem to have been inactively meandering or anastomosing; the latter, with multiple courses and wetlands between, have now all but disappeared from the scene. The situation on the Viennese Danube is similar, but on a higher energy level. Under the climatic and hydrological conditions of modern times, in its pre-channelisation state, the Viennese Danube section was a “gravel-dominated, laterally active anabranching river” associated with a “medium-energy, primarily non-cohesive floodplain” (according to the river/floodplain classification schemes of Nanson and Knighton 1996, and Nanson and Croke 1992). Such rivers show a complex channel network with numerous vegetated islands of different sizes and gravel bars. Examining historical sources with regard to whether the indicated riverine structures reflect natural fluvial processes or rather incorrect or generalised mapping is done by undertaking comparison with the potential forms and spatial extensions of channel change and floodplain evolution. On the Viennese Danube, the highly variable alpine flow regime with high loads of coarse bed material is one main underlying factor. Prior to channelisation, c. 500,000 m^3^ gravel and 5.6 million tons suspended load were transported annually down the Danube (Penck 1891; Schmautz et al. 2000).

In Vienna, summer and autumn floods after heavy rainfalls in the upper catchment, thaw floods in spring and the very typical ice jam floods in winter were the main reasons of sudden channel changes. This was especially true when ice jams suddenly disintegrated. Due to the high bed shear stress, new channels incised into the floodplain terrain (first order avulsion) or led to the reoccupation of abandoned arms or crevasse channels (second order avulsion; Richards et al. 1993). At side arms, large woody debris—originally a typical phenomenon with the unregulated Danube—had similar consequences. It is plausible that centuries of timber harvesting in the floodplains anthropogenically reduced the development of large woody debris compared to hypothetical ‘natural’ conditions. Besides channel changes caused by severe floods, flows between mean water and bankfull water level (approx. 1-year flood) contributed to lateral channel migration, which could amount to an average of 25 m per year at cut banks (Hohensinner, unpublished). Since some side arms developed into meander bends, meander cut-offs also occurred; this led to the accretion of the abandoned channel.

Due to the different forms of channel adjustments and floodplain inundation—active overflow, backwater flooding, or seepage inundation—such floodplains featured a great variety of depositional processes. Lateral point-bar accretion, overbank vertical-deposition, braid-channel accretion in wider profiles and abandoned channel accretion were most typical. The different processes are associated with specific sediment fractions. Annual erosion rates ranged from 1.6 % of the floodplain terrain in the Lobau directly downstream from Vienna to 2.5 % in the more dynamic Danube sections such as the Machland, 160 km upstream from Vienna (Hohensinner and Jungwirth 2009). Within a few decades, large shares of the floodplain terrain were renewed. Accordingly, high shares of morphologically young terrain were typical.

Such information and the experience from other riverscape reconstructions (Machland, Lobau and *Alluvial Zone National Park* downstream from Vienna) allow a plausibility assessment of the riverine structures depicted in the various historical sources (Hohensinner et al. 2008, 2011; Hohensinner and Schuch 2008). Knowledge of the geomorphological and hydrological processes in addition allows predictions about potential morphological changes before and after the point in time depicted by a source.

### Historical river engineering measures

River engineering measures have changed the riverscape, in particular over the last 200 years. Consequently, research on historical hydraulic constructions is an integral component of GIS-based landscape reconstruction. General knowledge about the types, dimensions and durability of historical hydraulic constructions, and the estimation of their effectiveness and their potential impacts on the riverscape are both important. Until the early 19th century, wood was the primary construction material in Austria (Schemerl 1782; Pasetti 1859; Baumgartner 1862; Veichtlbauer 2010). The most simple bank protection measure was the placement of rows of wooden piles along the shoreline. Side arms were dammed up with hurdle works (*Flechtzäune*) consisting of branches from willows and alders growing nearby. As historical sources and literature indicate, both measures offered little resistance against fluvial dynamics, in particular against the shear stress of ice jams (Thiel 1904).^20^ Fascines (*Faschinen*), bundles of branches bound together (sometimes stuffed with stones), were more sophisticated hydraulic structures (Schemerl 1782; Pasetti 1859). They featured higher resistance and longer durability. Until the early 19th century, such fascine constructions were often arranged as spur dikes (*Buhnen* or *Sporne*) at more or less right angles to the river banks. While such constructions might have been suitable for lowland rivers, they were soon destroyed in an alpine river with ice jams occurring almost annually (Donau-Regulirungs-Commission 1868). At locations considered particularly important, stone constructions were already being used in the 16th and 17th centuries, partly in the form of caissons (*Senkkästen*). Such was the case at the inflow of the *Donaukanal* near Nußdorf (Hohensinner et al. 2013, in this issue) and at the banks of the *Donaukanal* close to the city walls (Thiel 1904).^21^ But even such solid constructions were repeatedly destroyed by ice jam floods and needed regular maintenance. From the late 18th century onwards, the construction method gradually changed from transverse (spur dikes) to longitudinal structures such as guiding walls and rip-rap. Improved transport facilities in the 19th century, allowed wood to be replaced with rock materials (Pasetti 1859, 1862; Klun 1863). These hydraulic constructions were more durable and could affect the development of the nearby riverscape more intensively than the wooden hydraulic constructions of earlier times.

To assess the potential impacts of river engineering measures on the riverscape, a database was compiled that integrates all hydraulic measures mentioned in written sources and historical literature or indicated in the topographical sources. During the project, almost 1,800 river engineering measures were identified, verified and localised as accurately as possible for the period from 1300 to 1950 CE. The duration of their existence was determined and integrated in the data set. This yielded a GIS-cadaster of historical regulation measures in and around Vienna. Since reports on historical flood damage can also provide valuable information about the former state of the river landscape and the hydraulic constructions, a register of such flood damage was also compiled. The analysis of both databases allows further conclusions to be drawn about the general dimensions, technical designs, and spatial and temporal clustering of historical river engineering measures.

### Using landmarks and data on historical bridges

Georeferencing techniques are commonly used for the rectification of aerial photographs or blueprints distorted due to changes in air humidity. The geographically correct positioning of incoherently projected historical maps and plans, however, calls for a more sophisticated approach. Landmarks that were stable over centuries provided the basis for georeferencing of various topographical sources with ArcGIS 10. This includes St. Stephen’s cathedral, parts of the city walls, the so-called *Lusthaus* in the *Prater* floodplain or road junctions in the northern suburbs of Floridsdorf and Aspern. Such stable landmarks cover in an optimal way the whole time span of the reconstruction (1529–2010) or at least several centuries. As such they constitute absolute landmarks. In contrast, relative landmarks existed for shorter time periods and have not remained until today. We determined their position relatively to the absolute ones; they typically served as reference points in reconstructing two or a few sequenced time situations. Most available landmarks fall into this category. Typically, these are landscape structures or human-built structures that exist for decades or for one or two centuries and vanish thereafter. Nevertheless, they provide valuable reference points for georeferencing. During the reconstruction process, as many relative landmarks as possible that could be used to establish spatial relationships between two or more subsequent time situations were identified.

Georeferencing of historical sources goes beyond typical landmarks, it includes (archaeological) findings of bridge remains and past hydraulic constructions. One example are the findings made during the great Danube regulation in Vienna in 1870–1875, when a new cut-off main channel was excavated. As described by Prokesch (1876) and Lederer (1876), extracting the 100- to 200-year-old hydraulic structures in the river bed near Nußdorf was a very challenging task. In total, a volume of about 163,000 m^3^ of old hydraulic structures, more than 18,000 running meters of ties (*Schwellen*) and thousands of wooden piles were extracted from the river bed and accurately mapped and described. Both authors drew partly incorrect conclusions as far as dating is concerned, but the GIS approach did allow the identification and spatial attribution of several hydraulic constructions indicated in plans from the 18th century. This enables accurate localisation of the findings encountered in the early 1870s.

**Fig. 5:**
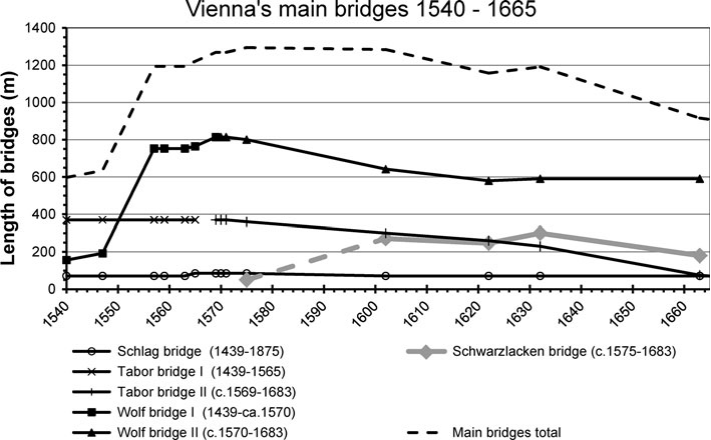
Main Danube bridges and bridge lengths in Vienna 1540–1665 (simplified chart, the bridge names refer to major shifts, e.g. *Tabor bridge* II in fact refers to several bridges constructed subsequently at approximately the same place; bridge lengths are interpolated between the known dates)

Historical descriptions of the lengths and locations of bridges are of equally high interest. For the GIS-reconstruction, we collected data on the lengths of the main bridges and changes in their length over time (Fig. 5).

The most important source is provided by Schmeltzl (1548), who documented the length of each bridge by counting the steps needed for crossing the bridge and the numbers of bridge pillars in 1547. He additionally noted the approximate distance between the outer and the inner main bridges. Bonifatius Wolmuet produced a map of the city of Vienna in the same year, for which he measured the length of the inner bridge (*Schlagbrücke*).^22^ Calculating Schmeltzl’s mean step length based on Wolmuet’s bridge length allows the lengths of the main bridges in 1547 to be calculated (for more details on the history of the Viennese bridges see Sonnlechner et al. 2013, in this issue). Since bridge length refers to bankfull width of a channel, which in Vienna coincides approximately with the 1-year flood, it provides a good measure for the discharge capacity of river channels. According to the “hydraulic geometry” approach introduced by Leopold and Maddock (1953), channel forms respond to changes in the flow regime. Bridge lengths beyond the range of widths typical for the Austrian Danube point either to inaccuracies in the cartographic sources or to amplified morphological turnover due to short-term channel shiftings (as happened in Vienna around 1565).

**Fig. 6:**
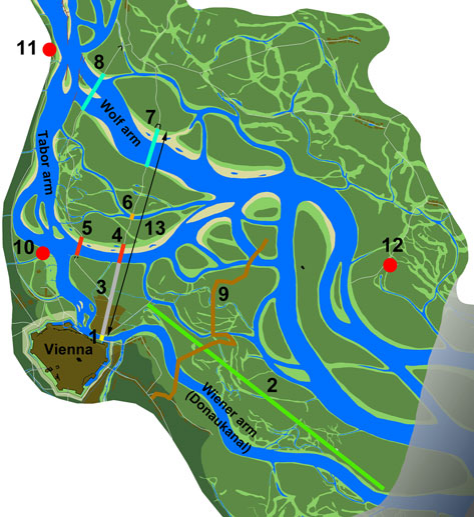
Main landmarks used for the reconstruction of the riverscape in 1570. *1* *Schlagbrücke*, *2* *Prater Main Avenue*, *3* historically surveyed transect from *Schlagbrücke* to *Tabor bridge*, *4* old *Tabor bridge* until 1565, *5* new *Tabor bridge* since 1569/70, *6* *Lackenbrücke*, *7* old *Wolf bridge* until 1565, *8* new *Wolf bridge* since 1569/70, *9* *Burgfriedsgrenze* with boundary stones, *10* confluence of tributary Alserbach, *11* village Nußdorf, *12* village Stadlau, and *13* distance between inner and outer bridge based on Schmeltzl 1547/48

Besides bridges, several other man-made features proved to be useful for georeferencing. One example is the *Prater Main Avenue* in the imperial hunting ground *Prater*. The originally 5.6 km long boulevard was constructed as a straight line in 1537/38 on a large island close to the city (Fig. 6). It functioned as a main landmark in historical riverscape cartography (Slezak 1980). Borders of land properties, hunting grounds or administrative borders also proved to be very useful; in particular, the so-called *Burgfriedsgrenze*, the jurisdiction border of the Viennese magistrate established in the Late Middle Ages that stretched far north into the floodplain (Opll 1986). It was marked with numerous stone boundary markers, the oldest going back to the 1540s, from which we know when they were set up (Opll et al. 1984). We assumed solid floodplain terrain at their locations at least for the time of their erection. Even if property borders were not directly marked in the maps they can be used as landmarks, because they are often indicated by different forms of land use.

Most of the topographical sources show the riverscape in the plan-view, but transect plans of the floodplain can also be extremely useful. In 1577, plans for the fortification of the *Unterer Werd* (later *Leopoldstadt*), the large island close to the city, gave rise to a topographical survey (Sonnlechner et al. 2013, in this issue). Starting from the inner bridge (*Schlagbrücke*), a transect across that island was surveyed ending with the *Tabor bridge* at the *Tabor arm*.^23^ Since the Danube had shifted the main flow to the northern *Wolf arm* at the latest in 1565/66, we assumed that the banks of the *Tabor arm* had remained largely stable since then. In combination with the known length of the *Tabor bridge,* the areal extents of a substantial part of the riverscape can be determined (Fig. 6).

Despite the described techniques, proper georeferencing is difficult to achieve for some historical sources. This mainly applies to older sources, where reference points can hardly be found; this holds true also for the correct positioning of riverine structures or hydraulic constructions at any site in the broad main Danube arm(s). In such cases, a workaround method based on several cartographic sources proved useful. The reference points are commonly located at the margins of the riverscape (settlements, roads, etc.), while the centre of the riverscape (main river arms) is difficult to deal with. Georeferencing two or three maps based on the available landmarks at the margins can at least limit the area of the potential site of the structure in question. The overall goal of georeferencing is to place each structure at the topographically correct position. In cases where such an absolute position of a structure cannot be exactly identified, it should at least show the same position across subsequent time situations. Otherwise the structure would unintentionally indicate a dislocation.

### The regressive-iterative GIS-reconstruction

In order to optimally incorporate the diverse data from the various historical sources into one model, we applied a dynamic regressive-iterative approach for the GIS-based reconstruction. Only if riverine structures, hydraulic constructions and infrastructure in the floodplain at different times are positioned exactly is it possible to discern causes and effects of change between different states of the riverscape. Knowledge about typical fluvial processes and the characteristics of past hydraulic constructions helps to better understand the alterations that are indicated in the sources.

**Fig. 7:**
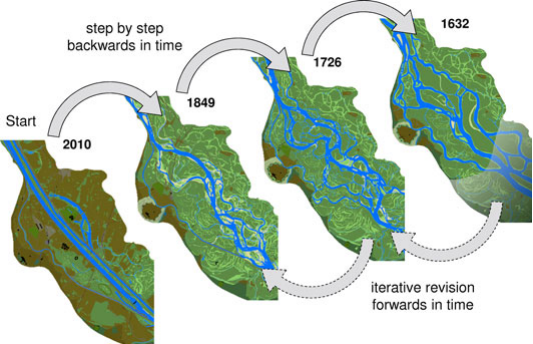
Schematic workflow of the regressive-iterative GIS-reconstruction of the historical river landscape

For the GIS, the current state of the Viennese river landscape served as a starting point. We reconstructed the ten historical states step-by-step backwards in time to the least known situation in 1529 (Fig. 7).

When we completed one of the situations (e.g. 1817), we started with the proximate older situation (1780) based on the completed one (1817). Every structure (GIS feature) of the 1817 riverscape was checked to determine whether it had remained unchanged, changed its appearance or vanished between 1780 and 1817. If any change was detected, we differentiated whether the change could derive from natural processes, from human interventions or was due to incorrect mapping. If a river arm shows an unexpected pattern compared to the former time situation that cannot be explained with typical channel forming processes, explanations had to be sought. One potential explanation concerns regulation measures, but most commonly inaccuracies of the plans and maps are the reason. In our example, the specific structure (GIS feature) of 1817 would be modified in accordance with the situation in 1780. When the reconstruction of the respective time situation (1780) was completed, we reviewed all information on the geographical structures (terrain topography/structures, infrastructure, etc.) to determine the extent to which it affected the interpretation of the structures in the more recent time situations. We had to clarify whether new conclusions on the state of the riverscape in the younger time situations needed to be drawn and corrections would have to be made. In most cases, not only the proximate younger situation (1817) had to be revised, but also the following ones (→1849 → 1875 → 1912 → 2010). Usually, the need for corrections decreases the closer one gets to the current state. We started with the reconstruction of the next situation (here: 1726) only after we had made the corrections in all relevant time situations.

Most sources focus on the riverscape close to the city, the *Wiener arm (Donaukanal)* and Nußdorf, while more remote areas are often depicted in less detail (e.g. remote river arms not shown in the sources). In such cases, we interpolated the position and pattern of the respective arm based on the time situations before and after, whereby typical forms of channel evolution and the occurrence and effects of major floods were considered. In several cases, written sources provided information about larger arms not shown in the maps (e.g. reports about flood damages on hydraulic constructions and bridges from the 16th century). Dead arms, vegetated ditches and terrain depressions in areas of the riverscape that clearly did not change over decades or even centuries are of specific interest. Such structures are mapped in great detail in the sources from 2010 back to 1849 (i.e. to the altitudinal survey from 1849), while they are poorly represented in the older sources. We work with the assumption that these features also existed earlier, as long as the respective area of the riverscape was not morphologically altered by active river arms that led to terrain erosion or aggradation. Copying such features back into previous situations in GIS is very helpful for the reconstruction. For example, we identified a long, vegetated ditch with some smaller backwaters in 1849 and 1817 that later proved to be the last remnant of the former *Fugbach* side arm in 1570 (compare Haidvogl et al. 2013 and Hohensinner et al. 2013, in this issue).

The regressive-iterative approach presented here is based on a permanent critical revision of the time situations already processed and ends only when the whole time series (back to 1529) is reconstructed. The further one goes back into the past and the more historical time situations are created, the more detailed and sound the more recent reconstructions also become.

## Synthesis

Depending on the source type, we encountered various problems when the sources were brought together during reconstruction. Especially in the case of the naturally dynamic riverine landscape, with its diverse and often short-lived structures (different types of water bodies, terrain features, etc.), maps were often created in a generalised, strongly simplified manner, or the cartographer omitted specific structures depending on the purpose of the respective map. Moreover, about half of the historical plans and maps show planned hydraulic structures, most of which were never implemented in the form shown. Today, the remains of 16th century to early 19th century hydraulic constructions no longer exist or were buried in the ground: the historical constructions cannot be verified in situ. Hence, the critical reading of sources is essential for the reconstruction process.

A synopsis of temporal changes or river morphological processes is difficult because the sources are different in type and usually show only fragmentary information. The GIS-based reconstruction method presented here yields a series of standardised maps that chronologically display altered states of fluvial landscapes. Based on the regressive-iterative GIS-technique described, the relevant information drawn from numerous written and topographical sources can be concentrated in a single dataset. The approach combines historical sources with information about typical fluvial processes and the potential impacts of past river engineering measures. This enables conclusions on the configuration of the riverscape even when information in the historical sources is patchy, spatially incorrect or otherwise unusable as such. The resulting dataset can be used for further spatio-temporal analysis, such as the identification of fluvial processes or the persistence of certain landscape elements. It does allow new insights and helps to detect dynamic fluvial processes and human-induced changes. One goal of the reconstruction method is to generate time series of maps, which foster communication of results to audiences beyond academia. Reconstruction is also a heuristic technique: during the process, one is forced to think about the historical development of each single structure. The accurate positioning by means of GIS reveals spatial inconsistencies relating to the analysed structures. Several descriptions and hypotheses in the older historical literature about the urban development of Vienna appeared conclusive. During the study, however, the integration of spatial information from the literature into the GIS revealed that either the described location or the assumed point in time could not be correct.

The method presented, as is the case with any method, has its limitations: some of the reconstructed structures cannot be positioned with certainty, but are on a particular spot on the map, potentially leading an observer to misinterpretations of the past reality. Since such inaccuracies are not primarily located close to settlement areas and involve more remote or highly dynamic areas of the riverscape, the potential misinterpretations are within reasonable bounds. One further downside must be mentioned: valuable information contained in the original sources that does not fit into the general design is lost due to data standardisation. The reconstruction is therefore no replacement for the original sources.

The resulting time series of historical states of the Viennese Danube riverscape in 1529, 1570, 1632, 1663, 1726, 1780, 1817, 1849, 1875, 1912 and 2010 provides a sound basis for interpreting the environmental conditions for Vienna’s urban development. It allows certain more or less stable features relevant for the history of Vienna to be localised and followed through time and thus puts history onto the map. The interdisciplinary approach clearly provided a major benefit in reconstructing the changes of the Viennese riverscape. The diverse approaches and findings of the historical and natural sciences (in this case, river morphology), provided vital synergies.

## Footnotes

1 Wien Museum, Topographische Sammlung, Sign. 48.068: Niclas Meldeman, “Der stadt Wien belegerung, wie die auff dem hohen sant Steffansthurn allenthalben gerings vm die gantze stadt zu wasser vnd landt mit allen dingen anzusehen gewest ist Vn von einem berumpten maeler…”, 1530.

2 Bayerische Staatsbibliothek München, Sign. Rar. 250: Caspar Stainhofer, “Gründtliche vnd khurtze beschreibung des alten vnnd jungen Zugs welche bede zu Einbeleittung… Kaiser Maximiliani des Anndern…”, 1566.

3 e.g. OeStA, AVA—FHKA, AHK, NOeHA, W 61/c/7/a (823), fol. 20r,v.

4 OeStA, AVA—FHKA, AHK, NOeHA, W 61/c/7/a (823), fol. 257–259.

5 Wien Museum, Topographische Sammlung, Sign. 95.961/1–3: “Wiener Donaulandschaft mit Darstellung einer zwischen dem Wiener Bürgerspital und dem Stift Klosterneuburg strittigen Au. Situation ca. 1570/80”, 1632; Sign. 95.961/4: “Detaillierte Darstellung der Wiener Donaulandschaft von 1632 mit Einzeichnung einer zwischen dem Wr. Bürgerspital und dem Stift Klosterneuburg strittigen Au sowie früheren Verläufen des Donauhauptstroms”, 1632; Stiftsarchiv Klosterneuburg, Sign. Sp. 379: “Mappa über die umliegenden Dörfer bey Wien”, 1632.

6 Wien Museum, Topographische Sammlung, Sign. 95.961/4.

7 OeStA, AVA—FHKA, AHK, NOeHA, N 27/b/3 (462), fol. 880–881: Hans Gast, 1598; OeStA, AVA—FHKA, Kartensammlung, Sign. F 245: Thomas Clausniez, 1601; OeStA, AVA—FHKA, AHK, NOeHA, N 27/b/3 (462), fol. 1116–1117: Thomas Clausniez, 1601; OeStA, AVA—FHKA, AHK, NOeHA, N 27/b/3 (462), fol. 1188–1190: Maximlian Saurer, 1601.

8 Except for Slezak (1980), who came to the same conclusion.

9 OeStA, KA, Kartensammlung, Sign. K VII e 152: Giuseppe Priami, “Abriß zu Wien zu Versicherung der Brükhen”, 1663.

10 OeStA, KA, Kartensammlung, Sign. B IX b 106: Leander Anguissola, “Grundt Riss des Donau Strom von dem Dorff Höfflein bis auf Wienn…”, 1688; Moravian Library (Brno, Czech Republic), Mollova mapová sbírka, Sign. Moll-0000.397: Max Anton Hoffmann von Anckherskron, Jacob Hoffmann and Jacob Hermandt, “Disse Mappa ist von der Löbl. Kays. Wasserbaues Commission untern Prasidio des Hoch und Wohlgebohrnen Herrn Herrn Carl Ferdinand des Heyl. Röm: Reichs Graff und Herr von Welz…”, 1700.

11 OeStA, KA, Kartensammlung, Sign. K VII e 152-5: Leander Anguissola, 1688/1712.

12 OeStA, KA, Genie-u. Plan-Archiv, Sign. C1/25, Env. A: Leander Anguissola and Johann Jacob Marinoni, “Vienna Austriae cum Suburbiis et adjacentibus Danubii Insulis …”, 1704; WStLA, Kartographische Sammlung, Sign. At 41: L. Anguissola and J. J. Marinoni, “Accuratissima Viennae Austriae Ichnographica Delineatio”, 1706.

13 OeNB, Kartensammlung, Sign. K I 98.480: J.J. Marinoni, “Neuer Atlas des Kayserl.en Wildban in Österreich unter der Ens”, 1726–1729.

14 Marinoni describes his improved survey technique in 1751 (Marinoni 1751).

15 OeStA, KA, Kartensammlung, Sign. B IX a 242: “Josephinische Landesaufnahme”, 1769–1785; OeStA, KA, Kartensammlung, Sign. B IX a 196-6: “Franziszeische Landesaufnahme”, 1806–1869.

16 NOeLB, Kartensammlung, Sign. B II 82: Christophorus de Lorenzo, “Nieder Oesterreichische Donau-Stromkarte”, surveyed 1816–1817, published 1819.

17 WStLA, Kartographische Sammlung, Sign. 2.2.3.2: “Franziszeischer Kataster”, 1817–1825/30.

18 OeNB, Kartensammlung, Sign. FKB 279-3, FKB 281-7: Florian Ritter v. Pasetti, Valentin Streffleur, Alexander Moering and Anton Dolezal, “Karte des Donau Stromes innerhalb der Gränzen des Österreichischen Kaiserstaates”, 1857–1867.

19 Technisches Museums Wien, Sign. L 20800: Valentin Streffleur and Carl Drobny, “Plastische Darstellung der Donau bei Wien nach der hydrotechnischen Vermessung vom Jahr 1849”, 1849; NOeLA, Regierungsarchiv, NOe Baudirection, Karton 494, Sign. Planschrank 10/Lade 7/III: Kazda and Nicolaus, “Lit. B: Plan des Donaudistrictes Wien”, 1849–1850; Magistrate of Vienna, MA 29, Archive, without Sign.: K. Kilian, “Lage- u. Schichtenplan des Donaugeländes bei Wien 1849”, K. Kilian, 1970s?

20 OeStA, AVA—FHKA, AHK, NOeHA, W 61/c/87/b (876), fol. 486–487.

21 OeStA, AVA—FHKA, AHK, NOeHA, W 61/c/87/b (876), fol. 605–695 and fol. 516.

22 Wien Museum, Topographische Sammlung, Sign. 31.021: M. Bonifatius Wolmuet, “Die fürstliche Stat wien in Osterreich wie Sy in Irem umbschwaif oder zarg beflossn. aus recht Geometruscher waß im grundt nidergelgt und …”, 1547; Wolmuet used “Werkschuh” as a measure of length, which refers only to 0.288 m compared to the imperial measure “Wiener Fuß” used in the late 18th and 19th centuries (=0.316 m; Wellisch 1898).

23 OeStA, KA, HKR, Sign. Exped 1579: O. Waldegara, Longitudinal section of the Untere Werd, 1577.

## References

[CR1] Aspinall R (2004). Modelling land use change with generalized linear models—a multi-model analysis of change between 1860 and 2000 in Gallatin Valley, Montana. Environ Manag.

[CR2] Baumgartner J (1862). Vorschlag zur Regulirung und Benützung der Donau bei Wien. Allg Bauztg.

[CR3] Behringer W (1999). Climatic change and witch-hunting: the impact of the Little Ice Age on mentalities. Clim Change.

[CR4] Bender O, Boehmer HJ, Jens D, Schumacher KP (2005). Using GIS to analyse long-term cultural landscape change in Southern Germany. Landsc Urban Plan.

[CR5] Bloch M (1931). Les caractères originaux de l’histoire rurale française.

[CR6] Clanchy MT (1993). From memory to written record: England 1066–1307.

[CR7] Cunfer G, Knowles AK (2008). Scaling the dust bowl. Placing history: how maps, spatial data, and GIS are changing historical scholarship.

[CR8] de Jager NR, Rohweder JJ, Nelson JC (2011) Past and predicted future changes in the land cover of the upper Mississippi river floodplain, USA. River Res Appl, early view (online)

[CR9] Donau-Regulirungs-Commission (1868) Bericht und Anträge des von der Commission für die Donauregulirung bei Wien ernannten Comités. Vorgetragen in der Plenarversammlung am 27. Juli 1868 und von derselben einstimmig angenommen. K.K. Hof- und Staatsdruckerei, Vienna

[CR10] Düriegl G (1980) Die Rundansicht des N. M. zur ersten Belagerung Wiens durch die Türken im Jahre 1529—Interpretation und Deutung. Wiener Schriften 44

[CR11] Foresman TW, Pickett STA, Zipperer WC (1997). Methods for spatial and temporal land use and land cover assessment for urban ecosystems and application in the greater Baltimore–Chesapeake region. Urban Ecosyst.

[CR12] Forschungsinitiative Umweltgeschichte, Ecker K, Grünweis FM, Müllner A, Sonnlechner C, Wilfing H, Winiwarter V (1999) Landschaft hat Geschichte. Historische Entwicklung von Umwelt und Gesellschaft in Theyern. CD-ROM, WUV, Vienna

[CR13] Francolin H, Hofhalter R (1561) Rerum praeclare gestarum intra et extra moenia munitissimae civitatis Viennensis, pedestri et equestri proelio, terra et aqua, elapso mense Junio anno Domini M.D.LX

[CR14] Girel J, Garguet-Duport B, Pautou G (1997). Landscape structure and historical processes along diked European valleys: a case study of the Arc/Isère confluence (Savoie, France). Environ Manag.

[CR15] Greco SE, Fremier AK, Larsen EW, Plant RE (2007). A tool for tracking floodplain age land surface patterns on a large meandering river with applications for ecological planning and restoration design. Landsc Urban Plan.

[CR16] Gurnell AM, Downward SR, Jones R (1994). Channel planform change on the River Dee meanders, 1876–1992. Regul Rivers Res Manag.

[CR17] Gurnell AM, Peiry J-L, Petts GE, Kondolf GM, Piégay H (2005). Using historical data in fluvial geomorphology. Tools in fluvial geomorphology.

[CR18] Haidvogl G (2008) Von der Flusslandschaft zum Fließgewässer: Die Entwicklung ausgewählter österreichischer Flüsse im 19. und 20. Jahrhundert mit besonderer Berücksichtigung der Kolonisierung des Überflutungsraums. Dissertation, University of Vienna

[CR19] Haidvogl G, Guthyne-Horvath M, Gierlinger S, Hohensinner S, Sonnlechner C (2013) Urban land for a growing city at the banks of a moving river: Vienna’s spread into the Danube island Unterer Werd from the late 17th to the beginning of the 20th century. Water Hist. doi:10.1007/s12685-013-0078-y 10.1007/s12685-013-0078-y PMC4811292 27069521

[CR20] Hegedüs Z, Duray B (2009). Landscape changes along the Tisza River in the southern Tisza Region of Hódmezövásárhely. Acta Climatologica et Chorologica Universitatis Szegediensis.

[CR21] Herrnegger M (2007) Historische Hydromorphologie und Geländetopografie der Wiener Donau-Auen. Master thesis, University of Natural Resources and Life Sciences, Vienna

[CR22] Hohensinner S (2008) Rekonstruktion ursprünglicher Lebensraumverhältnisse der Fluss-Auen-Biozönose der Donau im Machland auf Basis der morphologischen Entwicklung von 1715–1991. Dissertation, University of Natural Resources and Life Sciences, Vienna

[CR23] Hohensinner S, Jungwirth M (2009). Hydromorphological characteristics of the Danube River—the historical perspective. Z Österr Ing-Archit-Ver.

[CR24] Hohensinner S, Schuch M (2008) Naturversuch Bad Deutsch-Altenburg—Premonitoring Endbericht, Arbeitspaket B2b Landschaftsdynamik/Leitbild. via donau—Österreichische Wasserstraßen-Gesellschaft mbH, Vienna

[CR25] Hohensinner S, Herrnegger M, Blaschke AP, Habereder C, Haidvogl G, Hein T, Jungwirth M, Weiß M (2008). Type-specific reference conditions of fluvial landscapes: a search in the past by 3D-reconstruction. Catena.

[CR26] Hohensinner S, Jungwirth M, Muhar S, Schmutz S (2011). Spatio-temporal habitat dynamics in a changing Danube River landscape 1812–2006. River Res Appl.

[CR27] Hohensinner S, Lager B, Sonnlechner C, Haidvogl G, Gierlinger S, Schmid M, Krausmann F, Winiwarter V (2013) Changes in water and land: the reconstructed Viennese riverscape from 1500 to the present. Water Hist. doi:10.1007/s12685-013-0074-2 10.1007/s12685-013-0074-2 PMC4811290 27069520

[CR28] Howard AD, Malcolm G, Walling DE, Bates PD (1996). Modelling channel evolution and floodplain morphology. Floodplain processes.

[CR29] Kiss T, Fiala K, Sipos G (2008). Alterations of channel parameters in response to river regulation works since 1840 on the Lower Tisza River (Hungary). Geomorphology.

[CR30] Klun VF (1863). Flusskarten der Donau und der Theiss. Vortrag gehalten in der k.k. geographischen Gesellschaft am 28. Okt. 1862. Abh k.k. Geogr Ges.

[CR31] Knowles AK (2002). Past time, past place: GIS for history.

[CR32] Kondolf GM, Piégay H, Landon N (2007). Changes in the riparian zone of the lower Eygues River, France, since 1830. Landsc Ecol.

[CR33] Lederer I (1876). Zur Donau-Regulirung bei Wien. Die Beseitigung der alten Nussdorfer Stromwerke. Allg Bauztg.

[CR34] Leopold LB, Maddock T (1953) The hydraulic geometry of stream channels and some physiographic implications. Geological Survey Professional Paper 252, US Dept. of the Interior, Geological Survey, Washington, DC

[CR35] Lewin J (2010). Medieval environmental impacts and feedbacks: the lowland floodplains of England and Wales. Geoarchaeology.

[CR36] Macklin MG, Lewin J, Woodward JC (2012). The fluvial record of climate change. Philos Trans R Soc Assoc.

[CR37] Marinoni JJ (1751) De re ichnographica, cujus hodierna praxis exponitur, et propris exemplis pluribus illustrator. Austrian National Library, Vienna. http://openlibrary.org/books/OL24874525M/De_re_ichnographica

[CR38] Marston RA, Girel J, Pautou G, Piégay H, Bravard P, Arneson C (1995). Channel metamorphosis, floodplain disturbance, and vegetation development: Ain River, France. Geomorphology.

[CR39] Marti C, Bezzola GR (2004). Sohlenmorphologie in Flussaufweitungen. Mitteilungen der Versuchsanstalt für Wasserbau, Hydrologie u. Glaziologie der ETH Zürich.

[CR40] McCarney-Castle K, Voulgaris G, Kettner AJ, Giosan L (2011). Simulating fluvial fluxes in the Danube watershed: the “Little Ice Age” versus modern day. Holocene.

[CR41] Meldeman N (1530) Ein kurzer bericht uber die recht warhafftig Contrafactur Türckischer belegerung der stat Wien, wie dieselbig anzusehen unnd zuuersteen sey, welche zu rhum, preyß, lob und ehr ganzem Römischen Reich, gemeyner Ritterschaft, un in sonderheyt einem erbern Rath der stat Nürmberg, durch Niclas Meldeman yetz verfertigt, getruckt unnd außgangen ist. Nürnberg

[CR42] Mohilla P, Michlmayr F (1996). Donauatlas Wien: Geschichte der Donauregulierung auf Karten und Plänen aus vier Jahrhunderten. Atlas of the Danube River Vienna. A history of river training on maps and plans of four centuries.

[CR43] Nanson GC, Croke JC (1992). A genetic classification of floodplains. Geomorphology.

[CR44] Nanson GC, Knighton AD (1996). Anabranching rivers: their cause, character and classification. Earth Surf Process Landf.

[CR45] Opll F (1986) Alte Grenzen im Wiener Raum. In: Wiener Stadt-u. Landesarchiv, Ludwig-Boltzmann-Institut für Stadtgeschichtsforschung (eds) Kommentare zum Historischen Atlas von Wien 4, Vienna

[CR46] Opll F (2004) Wien im Bild historischer Karten. Die Entwicklung der Stadt bis in die Mitte des 19. Jahrhunderts. Böhlau Verlag, Vienna

[CR47] Opll F, Kopecky E, Putz HM (1984) 4.2.1/1 Grenzen im Wiener Raum/1—Stadt und weiteres Umland von der Römerzeit bis in die Mitte des 19. Jahrhunderts. Historischer Atlas von Wien, 2. Lieferung, Vienna

[CR48] Pasetti F (1859) Denkschrift der Donau-Regulirung bei Wien von der Kuchelau bis Fischamend. Manuscript, WStLA, Sign. 3.4.A.159, Vienna

[CR49] Pasetti F (1862) Notizen über die Donau-Regulirung im österreichischen Kaiserstaate bis zu Ende des Jahres 1861 mit Bezug auf die im k.k. Staatsministerium herausgegebene Übersichts-Karte der Donau. K.K. Hof- und Staatsdruckerei, Vienna

[CR51] Penck A (1891) Die Donau. Schriften Ver Verbreit naturwiss Kenntnisse in Wien 31, Vienna

[CR52] Pfister C (1980). The Little Ice Age: thermal and wetness indices for Central Europe. Interdiscip Hist.

[CR53] Pfister C (2007). Climatic extremes, recurrent crises and witch hunts: strategies of European societies in coping with exogenous shocks in the late sixteenth and early seventeenth centuries. Mediev Hist.

[CR54] Pisút P (2002). Channel evolution of the pre-channelized Danube River in Bratislava, Slovakia (1712–1886). Earth Surf Process Landf.

[CR55] Prokesch A (1876). Die alten Nußdorfer Wasserbauwerke. Bl Ver Landeskd Niederösterr N. F..

[CR56] Richards K, Chandra S, Friend P, Best JL, Bristow CS (1993). Avulsive channel systems: characteristics and examples. Braided rivers.

[CR57] Schemerl J (1782). Abhandlung über die vorzüglichste Art an Flüssen und Strömen zu bauen.

[CR58] Schmautz M, Aufleger M, Strobl T (2000). Wissenschaftliche Untersuchung der Geschiebe- und Eintiefungsproblematik der österreichischen Donau.

[CR59] Schmeltzl W (1548) Ein Lobspruch der Hochlöblichen weitberümbten Khünigklichen Stat Wien in Osterreich, wölche wider den Tyrannen vnd Erbfeindt Christi nit die wenigist, sondern die höchst Hauptbefestigung der Christenhait ist, Rö. Khü. May. &c. vnserm aller genedigsten Herrn zu Ehren beschriben/durch Wolffgang Schmeltzl, reprint from 1849, Kuppitsch, Vienna

[CR60] Schoor MM, Wolfert HP, Maas GJ, Middelkoop H, Lambeek JJP, Marriott SB, Alexander J (1999). Potential for floodplain rehabilitation based on historical maps and present-day processes along the River Rhine, The Netherlands. Floodplains: interdisciplinary approaches.

[CR61] Schuppert C, Dix A (2009). Reconstructing former features of the cultural landscape near early Celtic princely seats in Southern Germany. A GIS-based application of large-scale historical maps and archival sources as a contribution to archaeological research. Soc Sci Comput Rev.

[CR62] Seebohm F (1883). The English village community examined in its relations to the manorial and tribal systems and to the common or open field system of husbandry: an essay in economic history.

[CR63] Skokanova H (2008). The impact of river engineering works on the Dyje River floodplain in the Czech Republic. Glob Environ.

[CR64] Slezak F (1977). Die italienischen Begründer der Wiener Donaukartographie. Der Donauraum, Z Donauforsch.

[CR65] Slezak F (1980). Wien und die frühe Donaukartographie. Stadtgeschichtsforschung und Kartenvergleich. Mitt Österr Geogr Ges.

[CR66] Sonnlechner C, Hohensinner S, Haidvogl G (2013) Floods, fights and a fluid river: the Viennese Danube in the sixteenth century. Water Hist. doi:10.1007/s12685-013-0077-z

[CR67] Stainhofer C (1566) Gründtliche vnd khurtze beschreibung des alten vnnd jungen Zugs welche bede zu Einbeleittung… Kaiser Maximiliani des Anndern… sampt derselben geliebsten Gemahl vnd Kindern von der Crönung von Franckfurt zu Wienn den 16. Martij richtet worden, sambt aller schönen vnd zierlichen Ehrenporten Prunnen vnd anderer Solenniteten warhafftigen angehaenckten Wienn. Bayerische Staatsbibliothek München, Sign. Rar. 250

[CR68] Suess E (1862) Der Boden der Stadt Wien nach seiner Bildungsweise, Beschaffenheit und seinen Beziehungen zum bürgerlichen Leben: eine geologische Studie. Vienna

[CR69] Thiel V (1904). Geschichte der älteren Donauregulierungsarbeiten bei Wien. I. Von den ältesten Nachrichten bis zum Beginne des XVIII. Jahrhunderts. Jahrb Landeskd Niederösterr.

[CR70] Veichtlbauer O (2010) Von der Strombaukunst zur Stauseenkette: Die Regulierung der Donau. In: Winiwarter V, Schmid M (eds) Umwelt Donau: eine andere Geschichte. Katalog zur Ausstellung des Niederösterreichischen Landesarchivs in Ardagger Markt 5. Mai-7. November 2010, Provincial Archive of Lower Austria, St. Pölten, pp 57–73

[CR71] Wellisch S (1898) Die Wiener Stadtpläne zur Zeit der ersten Türkenbelagerung. Z Österr Ing-Archit-Ver 50:537–541, 552–555, 562–565

[CR72] Winiwarter V (2006) Vom Glashaus zu Biosphere 2. Überlegungen zur totalen Kolonisierung von Natur. In: Dix A, Langthaler E (eds) Grüne Revolutionen. Agrarsysteme und Umwelt im 19. und 20. Jahrhundert. Jahrb Gesch ländl Raumes, Innsbruck, pp 199–215

[CR73] Wolfert HP (2001) Geomorphological change and river rehabilitation: case studies on lowland fluvial systems in the Netherlands. Alterra Scientific Contributions 6, Wageningen

[CR74] Wünsch J (1914) der Einzug Kaiser Maximilians II. in Wien 1563. Ber Mitt Altertums-Ver zu Wien, Bd. XLVI & XLVII, Jg. 1914:9–34

